# Potential Roles of the Free Salivary Microbiome Dysbiosis in Periodontal Diseases

**DOI:** 10.3389/fcimb.2021.711282

**Published:** 2021-09-22

**Authors:** Jing Diao, Chao Yuan, Peiyuan Tong, Zhangke Ma, Xiangyu Sun, Shuguo Zheng

**Affiliations:** ^1^Department of Preventive Dentistry, Peking University School and Hospital of Stomatology, National Center of Stomatology, National Clinical Research Center for Oral Diseases, National Engineering Laboratory for Digital and Material Technology of Stomatology, Beijing, China; ^2^Department of Stomatology, Peking University Third Hospital, Beijing, China; ^3^Department of Paediatric Dentistry, School & Hospital of Stomatology, Tongji University, Shanghai Engineering Research Centre of Tooth Restoration and Regeneration, Shanghai, China

**Keywords:** salivary microbiome, gingivitis, generalized periodontitis (stage I/II), non-surgical therapy, dysbiosis, full-length 16S rRNA gene sequencing

## Abstract

Saliva is a vital mediator in the oral cavity. The dysbiosis of free bacteria in saliva might be related to the onset, development, prognosis, and recurrence of periodontal diseases, but this potential relationship is still unclear. The objective of this study was to investigate the potential roles of the free salivary microbiome in different periodontal statuses, their reaction to nonsurgical periodontal therapy, and differences between diseased individuals after treatment and healthy persons. We recruited 15 healthy individuals, 15 individuals with gingivitis, and 15 individuals with stage I/II generalized periodontitis. A total of 90 unstimulated whole saliva samples were collected and sequenced using full-length bacterial 16S rRNA gene sequencing. We found that as the severity of disease increased, from healthy to gingivitis and periodontitis, the degree of dysbiosis also increased. A higher abundance of *Prevotella intermedia* and *Catonella morbi* and a lower abundance of *Porphyromonas pasteri, Prevotella nanceiensis*, and *Haemophilus parainfluenzae* might be biomarkers of periodontitis, with an area under curve (AUC) reaching 0.9733. When patients received supragingival scaling, there were more pathogens related to recolonization in the saliva of periodontitis patients than in healthy persons. Even after effective nonsurgical periodontal therapy, individuals with periodontitis displayed a more dysbiotic and pathogenic microbial community in their saliva than healthy individuals. Therefore, the gradual transition in the entire salivary microbial community from healthy to diseased includes a gradual shift to dysbiosis. Free salivary pathogens might play an important role in the recolonization of bacteria as well as the prognosis and recurrence of periodontal diseases.

## Introduction

Periodontal diseases refer to various pathologies that occur in periodontal tissues, among which gingivitis and generalized periodontitis are the most common forms (Caton et al., 2018; Chapple et al., 2018). Gingivitis is an early inflammatory condition of the gums. If left untreated, it results in progressive destruction of the periodontal supporting tissues and clinically detectable attachment loss, establishing periodontitis (Newman et al., 2015). Periodontitis is one of the leading causes of tooth loss, endangering patients’ general and mental health and imposing high economic costs (Petersen and Ogawa, 2012). It has also been proven to be associated with various systemic diseases, such as diabetes (Preshaw et al., 2012) and Alzheimer’s disease (Dioguardi et al., 2020). If periodontitis is not well controlled, periodontal inflammation negatively affects glycemic control (Mauri-Obradors et al., 2018). According to the results of the 4th National Oral Health Epidemiological Survey of China, the percentage of subjects with healthy periodontal conditions was less than 10% among 35- to 45-year-old Chinese adults (Sun et al., 2018). The situation worsens with aging. The global age-standardized prevalence of severe periodontitis is static at 10-15% (Kassebaum et al., 2014). With an increasing life expectancy, periodontitis has predictably caused a growing burden worldwide. The severe impact and universality of periodontal disease necessitate investigation of early diagnosis and risk assessment.

The subgingival microbiota is considered to be the initiator of periodontal disease (Socransky et al., 1998). Simultaneously, the technique of using subgingival plaque or gingival crevicular fluid as a sample to evaluate periodontal status is complex and not convenient for extensive community-wide periodontal disease screening. Therefore, the use of saliva as a diagnostic tool for oral or systemic diseases has attracted attention because it is simple, fast, and noninvasive to collect whole saliva (Yoshizawa et al., 2013; Nunes et al., 2015). A previous study demonstrated the possibility of using at-home self-collected saliva samples as an adequate alternative for SARS-CoV-2 detection (Braz-Silva et al., 2020). Certain bacteria in saliva might have the potential to represent biomarkers of some systemic diseases, such as gestational diabetes mellitus (Li et al., 2021).

Saliva is an important mediator in the oral cavity. Studies have found a correlation between subgingival and salivary levels of specific bacteria present in periodontal tissues in patients with periodontitis (He et al., 2012; Haririan et al., 2014; Belstrøm et al., 2017). Meanwhile, it was reported that bacteria from the untreated diseased sites of individuals with periodontitis recolonized in the treated areas *via* saliva (Chen et al., 2018). These results imply that bacteria can freely disseminate to any oral site *via* saliva. Therefore, free periodontal disease-associated taxa in saliva might play an important role in the development and prognosis of periodontal diseases.

To date, many studies have focused on the salivary periodontal disease-associated microbiota in healthy and diseased individuals (Salminen et al., 2014; Salminen et al., 2015; Belstrøm et al., 2016; Damgaard et al., 2019; Lundmark et al., 2019) as well as the changes that occur before and after nonsurgical periodontal therapy (Komaki et al., 2017; Belstrøm et al., 2018; Chen et al., 2018). Most of these studies used quantitative real-time polymerase chain reaction (qPCR) or partial variable regions of the 16S rRNA gene for sequencing, making it impossible to obtain accurate information at the species level (Singer et al., 2016). Some bacteria of the same genus were associated with both disease and health, suggesting different pathogenic potentials of species within the same genus (Chen et al., 2018). Thus, it is of great significance to deeply sequence these organisms at the species level. In addition, the selection of the 16S rRNA gene sequence regions might bias the results (Cruaud et al., 2014; Tremblay et al., 2015). Based on third-generation PacBio sequencing technology, studies have shown that full-length bacterial 16S rRNA gene sequencing can provide accurate information at the species level and detailed identification of complex microbial communities with high throughput (Singer et al., 2016; Wagner et al., 2016; Johnson et al., 2019).

Therefore, we examined the salivary microbiota in periodontally healthy individuals (n=15) and in individuals with gingivitis (n=15) or generalized periodontitis (stage I/II) (n=15) before and after nonsurgical periodontal therapy using full-length bacterial 16S rRNA gene sequencing. We aimed to reveal the potential roles of the free salivary microbiota in different periodontal statuses and their reaction to treatment to determine the onset, development, and prognosis of salivary microbiome dysbiosis in persons with periodontal diseases. These results may offer a foundation for developing effective treatment strategies and helping in the assessment of prognosis.

## Materials and Methods

### Ethics Approval and Informed Consent

This study was ethically approved by the Peking University Biomedical Ethics Committee (issuing number: IRB00001052-16072). All participants signed the informed consent before enrollment.

### Subjects and Design

**Figure 1:**
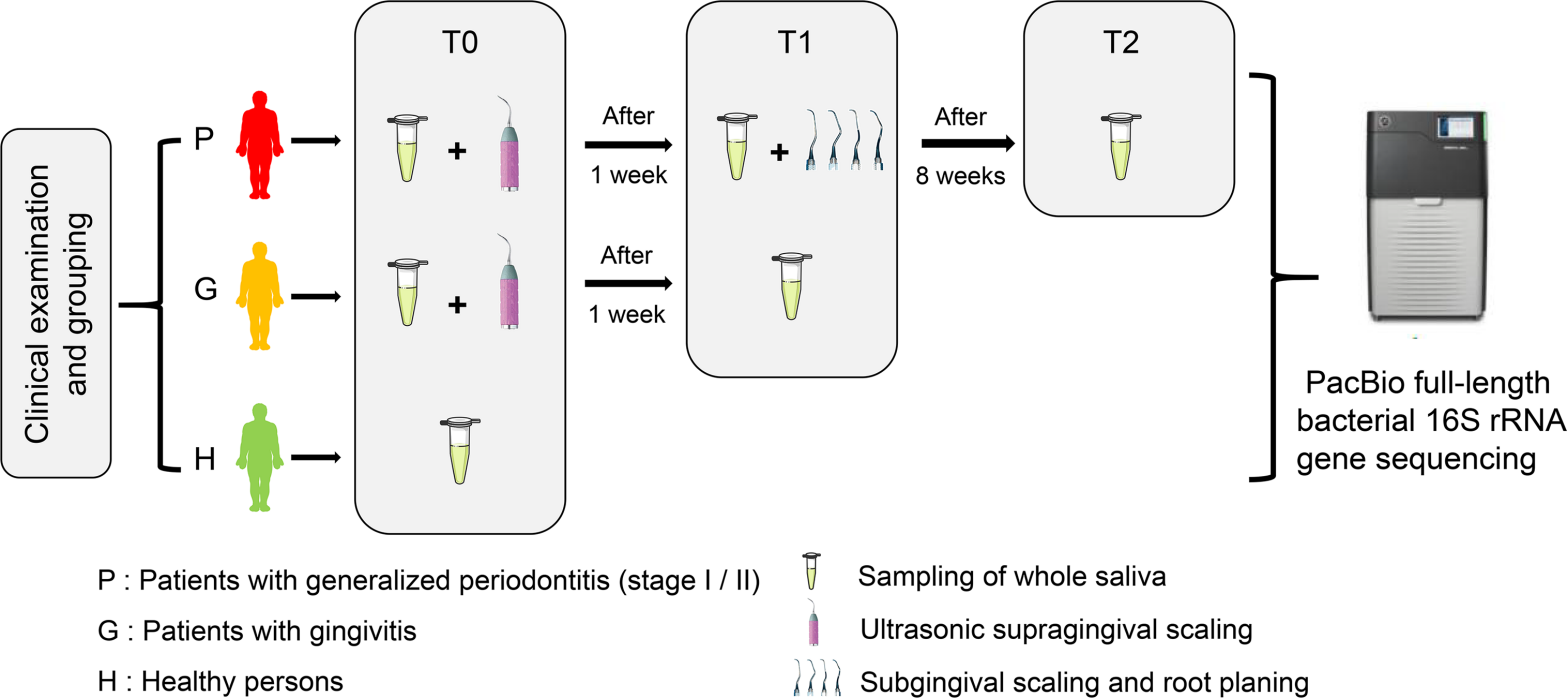
The flow chart of this study showing the enrollment and classification of participants to subsequent microbiota analyses.

A total of 90 unstimulated whole saliva samples were obtained from 15 periodontally healthy adults (H), 15 adults diagnosed with gingivitis (G), and 15 adults with generalized periodontitis (stage I/II) (P), all of whom were recruited from the Peking University School of Stomatology from March to September 2017. The flowchart of this study is shown in **Figure 1**. At baseline (T0), participants were enrolled and classified after reviewing their medical and dental history and oral clinical examinations. Clinical examinations of all participants were performed by one specialized dentist.

The inclusion criteria of the present study were as follows: (1) older than 20 years, (2) at least 20 naturally remaining teeth (excluding third molars), and (3) systemically healthy. The exclusion criteria were: (1) received periodontal treatment in the last 12 months, (2) used antibiotics or immunosuppressant medication within 3 months, (3) were pregnant or lactating, (4) were current or former smokers, (5) were diagnosed with systemic disease or disease/infection that may affect periodontal health status (e.g., diabetes), (6) wearing orthodontic appliances, (7) oral mucosal inflammation, or (8) severe untreated dental caries.

All participants were intergroup matched by sex and age as much as possible before the study commenced. All participants were sampled at baseline, and then groups G and P received ultrasonic supragingival scaling treatment. One week later (T1), groups G and P were sampled again, and then group P received subgingival scaling and root planning therapy using hand instruments (Gracey curettes 5/6, 7/8, 11/12, 13/14). Eight weeks after the completion of subgingival scaling and root planning treatment (T2), only group P was sampled for reevaluation. All participants received a full-mouth dental examination each time after sampling. During the intervals between sampling, there was no antibiotic medication or use of other drugs possibly affecting the microbiota composition, while no clinically significant condition emerged in this period as well. All periodontal treatments were performed by residents of the Department of Preventive Dentistry, Peking University Hospital of Stomatology. The quality of treatment and accuracy of clinical measurement were inspected by a clinical instructor. The specific clinical periodontal parameters are supplied in **Appendix Tables 1, 2**.

### Clinical Examination and Classification

Clinical examinations of all participants were performed by one specialized dentist using manual periodontal probes (PCPUNC 15; HuFriedy Mfg. Co., Inc., Chicago, IL, USA). The full mouth plaque scores of subjects were recorded. The clinical periodontal indices, including probing depth (PD), bleeding on probing (BOP), and bleeding index (BI), were measured at six sites per tooth (mesio-buccal, mid-buccal, disto-buccal, mesio-lingual, mid-lingual, and disto-lingual). PD was measured to the nearest scale.

Participants were classified into the group of generalized periodontitis (Stage I/II) (the P group), gingivitis (the G group), and periodontally healthy control (the H group) based on their periodontal status in accordance with the clinical criteria stated in the consensus report of the World Workshop in Periodontitis (Caton et al., 2018; Chapple et al., 2018). The generalized periodontitis (Stage I/II) group (n=15) included individuals with at least 30% of teeth with CAL ≥ 1 mm and PD ≥ 4 mm. The G group (n=15) exhibited no CAL, no site with PD > 3 mm, BOP > 20%, and no radiographic alveolar bone loss. The H group (n=15) exhibited no sites with attachment loss, no sites with PD > 3 mm, BOP ≤ 20%, and no radiographic alveolar bone loss.

### Saliva Sampling and Total Genomic DNA Extraction

All participants were asked not to eat or use oral hygiene methods for at least two hours until sampling. Before collection, each participant was asked to rinse their oral cavity with water and then rest for 10 min. Two milliliters of unstimulated whole saliva were collected at 8:00 – 9:00 a.m. and then transferred to the laboratory on ice as soon as possible and centrifuged at 10,000 × g for 10 min at 4°C. The supernatant was removed, and the precipitate was stored at -80°C before DNA extraction.

Microbial DNA extraction was performed using the QIAamp DNA Mini Kit (Qiagen, Hilden, Germany), following the manufacturer’s instructions. Concentration and purity testing of the DNA was performed using a NanoDrop 8000 spectrophotometer (NanoDrop Technologies, Wilmington, DE, USA). The integrity of bacterial genomic DNA was checked by 1.20% agarose gel electrophoresis, and a negative control with only buffer was used. DNA samples were stored at -80°C until further use.

### PCR Amplification, Sequencing, and Quality Filtering

PCR amplification of the nearly full-length bacterial 16S rRNA genes was performed using the forward primer 27F (5’-AGAGTTTGATCMTGGCTCAG-3’) and the reverse primer 1492R (5’-ACCTTGTTACGACTT-3’). PCR amplification was performed under the following cycling conditions: initial denaturation 98°C 2 min, denaturation 98°C 15 s, annealing 55°C 30 s, extension 72°C 30 s, final extension 72°C 5 min, and 10°C hold for 25-30 cycles. Full-length 16S rRNA gene sequencing was conducted using the PacBio Sequel platform (Shanghai Personal Biotechnology Co., Ltd, Shanghai, China). Sequences were quality filtered *via* Vsearch (v2.13.4_linux_x86_64) using the fastq_filter command and selecting the fastq_maxee parameter to discard sequences with more than the specified number of expected errors (Rognes et al., 2016).

### Data Processing and Statistical Analysis

Analysis of sequencing data was primarily performed using the QIIME2 platform and R package (3.5.0). The data were further analyzed as follows: (1) differences in age and sex were compared using t-tests and χ2 tests; (2) rarefaction curves, taxonomic composition maps at the species level, and Venn diagrams were constructed; (3) the Chao1 index, Shannon index, and Simpson index were calculated among the baseline groups; (4) principal coordinates analysis (PCoA) based on Bray-Curtis distance was used to examine community differences at baseline; (5) interindividual variation based on weighted UniFrac distance was compared by Kruskal–Wallis test *via* GraphPad Prism 8 (GraphPad, San Diego, California, USA); (6) relative abundance differences in bacterial taxa between groups H and P0 were compared by Welch’s t-test using STAMP (v2.1.3) (Parks et al., 2014). (7) receiver operating characteristic (ROC) analysis and analysis of clinical parameters were conducted using SPSS version 23.0 (IBM, Armonk, NY, USA); (8) the random forest algorithm was constructed to rank the importance of all operational taxonomic units (OTUs) and show their abundance; (9) MetagenomeSeq analyses were used to identify OTUs with statistically significant differences between groups; and (10) functional prediction of microbiota was conducted on PICRUSt 2 (Phylogenetic Investigation of Communities by Reconstruction of Unobserved States). The pathway abundances were predicted based on KEGG orthologs. *P* < 0.05 was considered statistically significant, and all *P*-values were two-sided.

### Data Availability

The raw sequencing data of this study are available in the NCBI Sequence Read Archive under accession number PRJNA725813.

## Results

### Characteristics of Study Subjects

The P group included 15 patients with generalized periodontitis (stage I/II) (mean age 39.80 ± 11.08 years, male =6, female=9). The G group included 15 patients with gingivitis (mean age 33.67 ± 11.20 years, male =5, female=10), and the H group included 15 periodontally healthy individuals as controls (mean age 33.80 ± 11.30 years, male =5, female=10). There were no significant differences in the mean age (P *vs*. H: *P* = 0.153; G *vs*. H: *P* = 0.974) or sex distribution (*P* = 0.908) among the selected groups.

### The Composition and Diversity of Salivary Microbiota

A total of 1,214,934 sequences were generated after quality filtering, with an average of 13,499 (range from 7,449 to 17,582) sequences per sample. Most of the sequences were 1420-1490 bp in length. The species richness of the salivary microbiota of each sample was estimated by rarefaction analysis (**Appendix Figure 1**). The overall structure of the salivary microbiota by species in different groups of saliva samples is shown in **Appendix Figure 2**. The legend shows the top 50 taxa in terms of mean relative abundance. The number of shared and unique OTUs in each group is shown in **Appendix Figure 3**.

**Figure 2:**
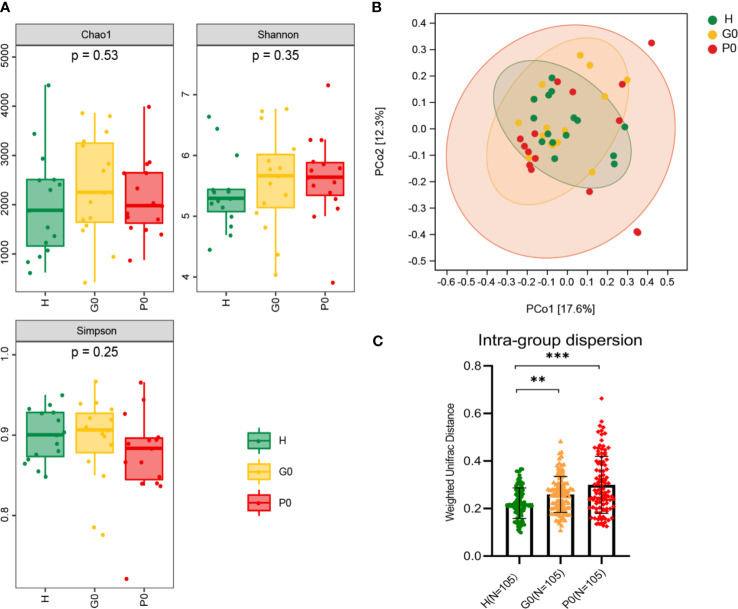
Comparisons of alpha diversity and beta diversity among the three baseline groups, H, G0, and P0. **(A)** Microbial richness presented by Chao1 and microbial diversity presented by Shannon and Simpson indices. **(B)** PCoA based on Bray-Curtis distance exhibiting the variation of community structure in the three groups. **(C)** Intragroup dispersion presented by the corresponding interindividual weighted UniFrac distances for each group. **P < 0.01, ***P < 0.001 by Kruskal-Wallis test. Each color represents one group: green for H, yellow for G0, and red for P0.

The α-diversity indices of the baseline groups, including the Chao 1 index, Shannon index, and Simpson index, are displayed in **Figure 2A**. We found that the Chao1 index in the H group was the lowest, and it was the highest in the G0 group, which was similar in the Shannon index. The Simpson index in the P0 group was the lowest, and it was the highest in the G0 group. However, there were no significant differences among any of these comparisons.

β-Diversity, shown by principal coordinates analysis (PCoA) of Bray-Curtis distance, was analyzed to determine the salivary microbial community structure at baseline (**Figure 2B**). The closer the distance between samples shown in the diagram, the more similar the microbial community structure was. The results showed that the salivary microbial community structure was similar among the three groups. At the same time, the intragroup dispersion was more significant in the P0 group than in the other two groups.

Then, we extracted the corresponding interindividual weighted UniFrac distances of the three baseline groups (**Figure 2C**). The interindividual distances in group H were significantly lower than those in groups G0 and P0 (*P*=0.002, *P*<0.001, respectively) by the Kruskal-Wallis test.

### Marker Species Analysis Between Groups H and P0

**Figure 3:**
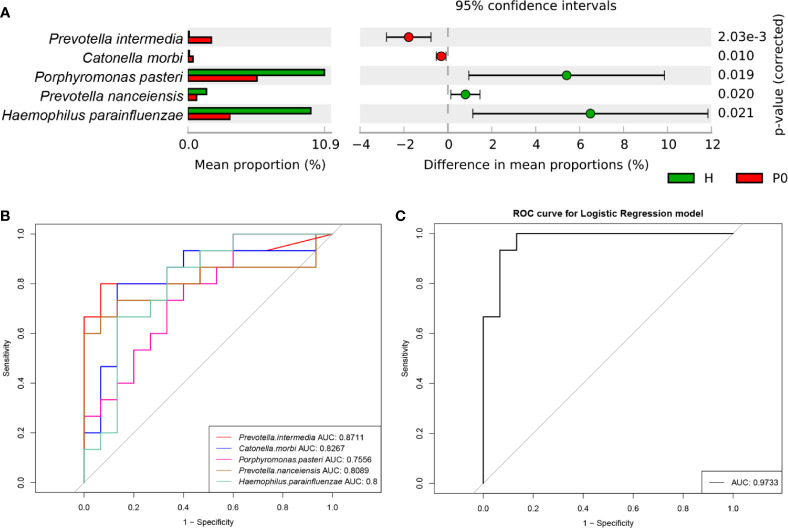
Differential species and ROC analysis. **(A)** Comparison of the relative abundance of bacteria by species between the H and P0 groups at baseline. *P* < 0.05 by Welch’s t-test. **(B)** Receiver operating characteristic (ROC) analysis of distinguishing group H from group P0 using detected species assessed by the area under the curve (AUC). **(C)** ROC curve for the logistic regression model of combined diagnosis by the five detected species.

Taxa with a median relative abundance below 0.1% were excluded. Then, the differential species between groups H and P0 were further investigated to identify specific microbial biomarkers that can be used to discriminate periodontitis. At the species level, a group of five species, *Prevotella intermedia, Catonella morbi, Porphyromonas pasteri, Prevotella nanceiensis*, and *Haemophilus parainfluenzae*, showed statistically significant differences in abundance between the H and P0 groups using Welch’s t-test (*P*<0.001, *P*=0.010, *P*=0.019, *P*=0.020, and *P*=0.021, respectively) (**Figure 3A**). *P*<0.05 was regarded as the threshold for statistical significance (two-sided).

Next, we conducted receiver operating characteristic (ROC) analysis to distinguish the H and P0 groups using the five species detected by comparing the relative abundance of predominant bacteria to construct classification models. Using *Prevotella intermedia, Catonella morbi, Porphyromonas pasteri, Prevotella nanceiensis*, and *Haemophilus parainfluenzae* as bacterial features, the model achieved optimal area under the curve (AUC) values of 0.8711, 0.8267, 0.7556, 0.8089, and 0.8, respectively (**Figure 3B**). When using the combination of the five species, the AUC value was as high as 0.9733 (**Figure 3C**).

### Group Classification Based on the Random Forest (RF) Algorithm

**Figure 4:**
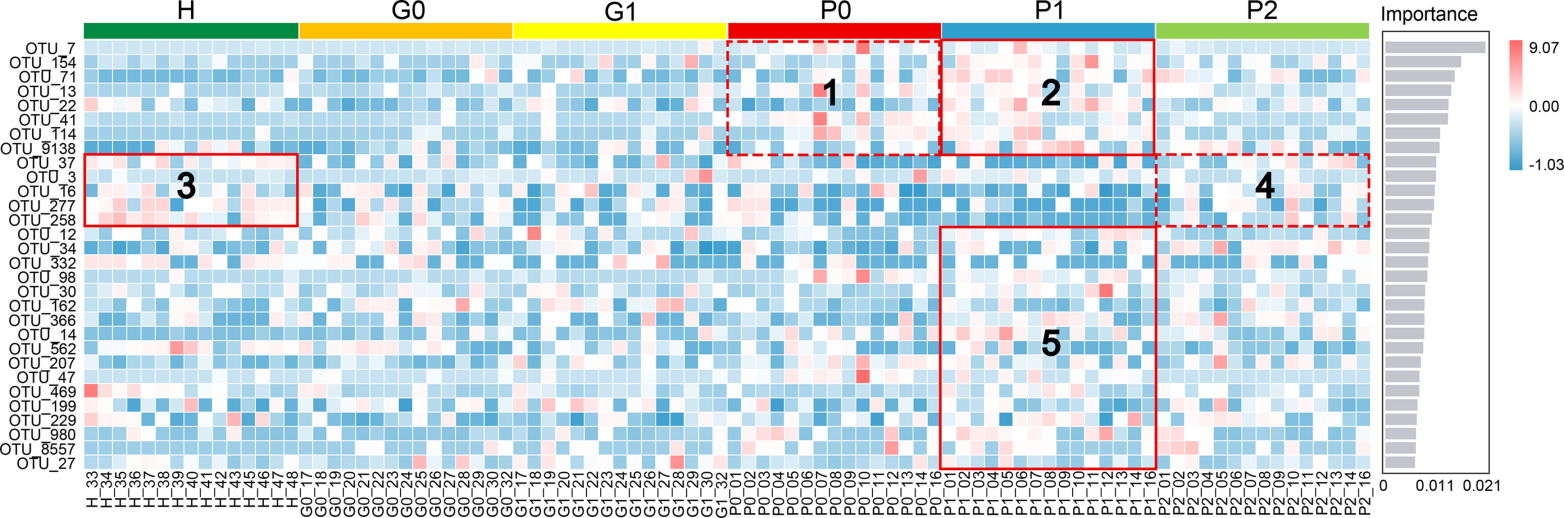
Random forest classification model for different periodontal statuses based on free salivary bacteria. The top 30 OTUs with the highest importance for classification are ranked on the left (see **Appendix Table 3** for the complete list of taxa), and the score of importance is shown on the right. Different colors on the top identify the six sample groups. The heat map shows the abundance distribution of these taxa in each sample. Boxes 1, 2, and 5 were characterized by high levels of disease-associated taxa. Boxes 3 and 4 were characterized by health-associated taxa and other bacteria considered to have low pathogenicity.

A classifier based on the random forest algorithm was constructed to rank the importance of all OTUs. The top 30 OTUs and their abundance information are shown in **Figure 4**. There were no significant differences in the abundance of OTUs between groups G and H. Box 1 and Box 2 were characterized by high levels of periodontal disease-associated taxa, such as *Porphyromonas gingivalis, Streptococcus gordonii, Prevotella nigrescens, Porphyromonas endodontalis, Filifactor alocis*, and *Tannerella forsythia*, corresponding to groups P0 and P1, respectively (Socransky et al., 1998; Holt and Ebersole, 2005; Bedran et al., 2012; Aruni et al., 2014; Zhang et al., 2017; Edmisson et al., 2018). Interestingly, a higher abundance of disease-associated taxa was observed in group P1 (Box 2) than in group P0 (Box 1). This was similar in Box 5. Box 3 and Box 4 were characterized by periodontal health-associated taxa and other bacteria considered to have low pathogenicity, such as *Prevotella nanceiensis, Lautropia mirabilis*, and *Prevotella melaninogenica* (Colombo et al., 2012; Teles et al., 2012; Aguilar-Durán et al., 2019; Ikeda et al., 2019; Beydoun et al., 2020; Papapanou et al., 2020). The abundance of health-related taxa was the highest in group H and the lowest in group P, and it was relatively high in group P2 among the three P groups.

### Differences at the OTU Level Before and After Treatment

**Figure 5:**
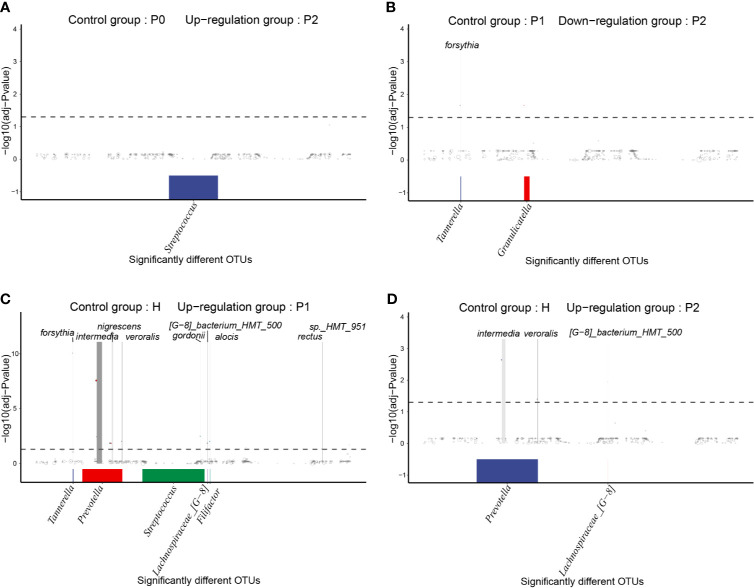
MetagenomeSeq analyses of significantly different taxa (occurrence frequency of OTUs in the upregulated group was greater than 0.3) between different groups. The differential taxa are annotated at the genus level at the bottom and at the species level at the top. **(A)** Upregulated OTUs in group P2 compared to P0. **(B)** Downregulated OTUs in group P2 compared to P1. **(C)** Upregulated OTUs in group P1 compared to H. **(D)** Upregulated OTUs in group P2 compared to H.

To determine differences in OTU levels before and after nonsurgical periodontal treatment, we conducted MetagenomeSeq analyses. There were no significantly different OTUs between groups P0 and P1, while the abundance of OTU_8637, which was annotated as *unclassified_Streptococcus*, was significantly increased in group P2 compared to P0 (**Figure 5A**). The quantities of OTU_114 and OTU_8431, annotated as *Tannerella forsythia* and *unclassified_Granulicatella*, respectively, were decreased in group P2 compared to P1 (**Figure 5B**).

Compared to group H, many different OTUs were increased in the P1 and P2 groups, most of which were periodontal disease-associated taxa. Interestingly, there were more types of significantly different taxa between groups P1 and H, including *Prevotella intermedia, Filifactor alocis, Tannerella forsythia, Prevotella melaninogenica, Prevotella nigrescens, Prevotella veroralis, Campylobacter rectus, Streptococcus gordonii, Lachnospiraceae [G-8] bacterium_HMT_500, Treponema* sp.*_HMT_951*, and *unclassified_Neisseria* (**Figure 5C**). After completing nonsurgical periodontal treatment, there were still enriched taxa, including *Prevotella intermedia, Lachnospiraceae_[G-8] bacterium_HMT_500, and Prevotella veroralis*, in group P2 *versus* group H (**Figure 5D**).

### Bacterial Functional Analysis and Comparison

Predictive function by PICRUSt2 based on the KEGG database showed that there were no statistically significant differences among any of the groups (**Appendix Figure 4**).

## Discussion

Gingivitis and generalized periodontitis are chronic infectious diseases derived from the dysbiosis of the subgingival microbiome (Hajishengallis and Lamont, 2012). Host inflammatory reactions play a vital role in their onset and development (Bartold and Dyke, 2013; Lamont et al., 2018). When the subgingival local bacterial community changes, the entire oral ecology is affected, and the free pathogens in saliva may reflect the degree of dysbiosis in the oral niche (Haririan et al., 2014; Belstrøm et al., 2016; Belstrøm et al., 2018; Chen et al., 2018; Damgaard et al., 2019; Lundmark et al., 2019). Therefore, saliva shows its importance here and offers a simple, noninvasive alternative to assist in the early diagnosis and risk assessment of periodontal diseases. This study demonstrated the free bacterial composition and potential roles in saliva in different periodontal statuses and their reaction to treatment using third-generation PacBio full-length 16S rRNA gene sequencing. Therefore, we gained more detailed and comprehensive information about microbial composition at the species level (Singer et al., 2016).

The cross-sectional comparison of α-diversity indices exhibited no statistically significant differences in baseline groups (**Figure 2A**). The PCoA based on Bray-Curtis distances (**Figure 2B**) indicates that the closer the distance between samples shown in the diagram, the more similar the microbial community structure was. Both results demonstrated that the salivary microbial community structure was similar among the three baseline groups. This is consistent with previous studies showing that the salivary microbiome primarily consists of bacteria shed from the surface of the mouth, especially from the tongue and throat (Segata et al., 2012; Krishnan et al., 2016). As the severity of disease increased, the interindividual distances based on weighted UniFrac distances became increasingly farther from healthy to gingivitis and periodontitis (**Figure 2C**). This might indicate that periodontal diseases are not driven by the same specific bacteria in different cases. There were varied compositions of pathogens in different individuals owing to their diverse susceptibility to bacteria. Then, the increase in the degree of dysbiosis led to a diseased status. The microbial community structure became increasingly dissimilar in individuals with periodontal diseases.

In the comparison between groups H and P0, we identified five bacterial species that showed a statistically significant difference in abundance (**Figure 3A**). According to their relative abundance, we assumed that a higher abundance of *Prevotella intermedia* and *Catonella morbi* and a lower abundance of *Porphyromonas pasteri, Prevotella nanceiensis*, and *Haemophilus parainfluenzae* might be characteristics of generalized periodontitis (stage I/II) compared to healthy conditions. A combination of these five bacteria at the species level might serve as a biomarker of generalized periodontitis (stage I/II) and help with rapid diagnosis, with an AUC reaching 0.9733 (**Figure 3C**). However, before application on a larger scale, this classifier requires validation in a larger population of different regions and races.

The results of the random forest algorithm (**Figure 4**) and the MetagenomeSeq analyses (**Figure 5**) offered us some information about the bacterial composition at the OTU level. Changes in free bacteria in the saliva after nonsurgical periodontal therapy were not as apparent as in subgingival plaques or gingival crevicular fluids in previous studies (Komaki et al., 2017; Belstrøm et al., 2018). There was no difference in OTUs between healthy individuals and persons with gingivitis at either T0 or T1. However, for patients with generalized periodontitis (stage I/II), the general tendencies were consistent with previous studies (Komaki et al., 2017; Belstrøm et al., 2018), including the increased abundance of health-associated taxa such as the *Streptococcus* genus and the decreased quantity of disease-associated taxa such as *Tannerella forsythia*.

Some of the different taxa between groups H and P1 were also found in other studies. One study found that *Campylobacter rectus* and *Prevotella nigrescens* in supragingival plaque and *Prevotella nigrescens* in subgingival plaque displayed increased proportions 4-7 days after professional cleaning (Teles et al., 2012). This result indicates that these taxa might be related to the recolonization of periodontal pathogens and the reestablishment of plaque biofilms. In addition, *Streptococcus gordonii* synergistically acts with *Porphyromonas gingivalis* to initiate biofilms on the tooth and provide binding sites for subsequent colonizing bacteria to attach and generate mature biofilms (Daep et al., 2011; Wright et al., 2013; Lamont and Hajishengallis, 2015; Kuboniwa et al., 2017). These results indicate that the enriched pathogens in the saliva of group P1 might be related to the reestablishment of plaque biofilms. Periodontitis patients who had undergone ultrasonic supragingival scaling a week prior and left their subgingival plaque unremoved experienced recolonization of periodontal pathogens.

Eight weeks after the end of nonsurgical periodontal treatment, periodontitis subjects’ clinical parameters improved significantly (**Appendix Table 1**). However, there were still enriched taxa in group P2 *versus* group H, including *Prevotella intermedia, Lachnospiraceae_[G-8] bacterium_HMT_500*, and *Prevotella veroralis*. *Prevotella intermedia* is a member of the “orange complex” (Socransky et al., 1998). The “orange complex” consisted of a tightly related core group including members of the *Fusohaderium nucleatum/periodoniicum* subspecies, *Prevotella intermedia*, *Prevotella nigrescens*, and *Peptostreptucoccus micros*. Species associated with this group included: *Eubacterium nodatum*, *Campylobacter rectus*, *Campylobacter showae*, *Streptococcus consteltatus*, and *Campylobacter graellis*. The species in this group were closely associated with one another, and this complex appeared closely related to the “red complex” and periodontal pocket depth (Socransky et al., 1998). *Prevotella veroralis* is usually found in adult patients with periodontitis (Rawlinson et al., 1993). According to these results, we found that it was impossible to change the free salivary bacteria in individuals with periodontitis into a totally healthy status if they simply underwent complete nonsurgical periodontal treatment one time. A previous study suggested similar results. The authors found that persons with well-maintained periodontitis exhibited a more dysbiotic subgingival microbial community than healthy persons (Lu et al., 2020). These results could help explain why chronic periodontitis is a rather stubborn disease which is easy to recur. Higher levels of pathogenic bacteria in the saliva might be one of the reasons for its recurrence.

In the functional analysis, we found no statistically significant difference among any of the groups (**Appendix Figure 4**). This might indicate that saliva is not a suitable medium for investigating functional changes in periodontal pathogenic bacteria. A limitation of the present study is that we did not sample subgingival plaques, which might be a better medium for metabolic analysis. We were also unable to investigate the relationship between subgingival plaque and free salivary bacteria. In addition, the periodontitis patients we included presented relatively mild clinical parameters. This might be a vital factor affecting functional analysis and other aspects of our research. Another limitation of our study is the limited sample size. The potential roles of the free salivary microbiome in periodontal diseases should be confirmed in a larger population with various periodontitis levels from mild to severe.

**Figure 6:**
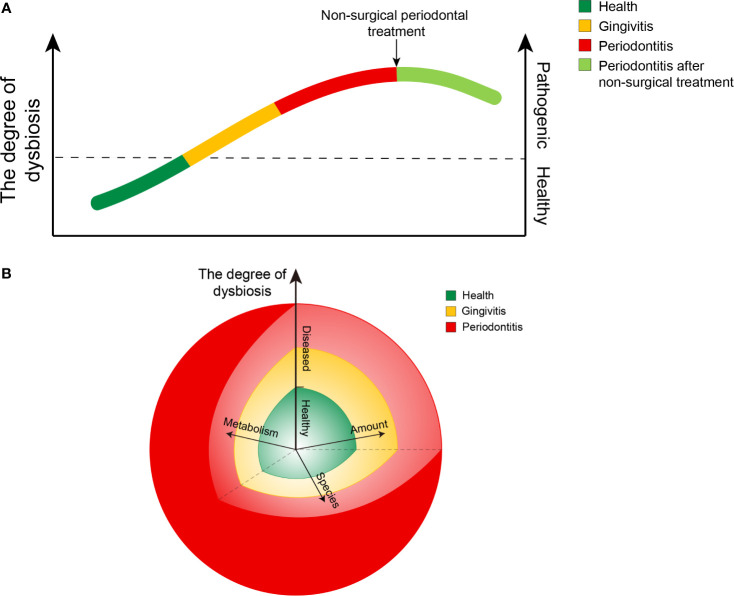
Diagrams of the ecological plaque hypothesis in periodontal disease. **(A)** Chart showing changing trends in the degree of dysbiosis from healthy to diseased and after nonsurgical treatment of periodontitis. **(B)** Chart showing changing trends in the degree of dysbiosis resulting from the combined action of three factors: the amount of bacteria, the species composition, and their metabolic function. The length of the arrows represents their respective influence. The angle between any two of the arrows represents an interaction between the factors, while the interaction is not yet well characterized.

From a healthy state to periodontitis, there was a gradual shift to dysbiosis, along with gingivitis (**Figure 6A**). This gradual transition in the entire microbial community from health to disease may be affected by several factors (**Figure 6B**). Under healthy conditions, the composition and metabolism of the microbial community may fluctuate within a small range, indicating a dynamic balance. Once the local microenvironment changes, the abundance of bacteria and the specific microbial composition of plaques may vary significantly (**Appendix Figures 2**, **3**). In addition, the metabolism of plaques may also be influenced, resulting in more pathogenic products. All of these transitions may accelerate the increase in the degree of dysbiosis, and the clinical status would eventually change from healthy to diseased. Once the individuals experience periodontitis, the free salivary bacterial community remains more dysbiotic and pathogenic than that in healthy individuals, even though they were administered nonsurgical periodontal treatment (**Figure 6A**). Most of the current therapeutic interventions aim to remove local plaque and its products to improve the local microenvironment to a healthy state. Few treatment strategies have taken pathogenic bacteria in the saliva into account. A longitudinal design is needed to explore the specific role of free salivary bacteria in the relapse in patients with periodontitis after nonsurgical periodontal treatment.

The results of our study help us better understand the ecological plaque hypothesis and provide inspiration for treatment strategies. First, the gradual transition in the entire salivary microbial community from healthy to diseased involved a gradual shift to dysbiosis. Timely and effective intervention in gingivitis is necessary to prevent the further development of dysbiosis. Second, free salivary pathogens might play an important role in the recolonization of bacteria and the prognosis and recurrence of periodontal diseases. Periodic periodontal maintenance seems essential to reduce the abundance of free pathogens in the saliva.

## Data Availability Statement

The raw sequencing data of this study are available in the NCBI Sequence Read Archive with the accession number PRJNA725813.

## Ethics Statement

The studies involving human participants were reviewed and approved by Peking University Biomedical Ethics Committee (issuing number: IRB00001052-16072). The patients/participants provided their written informed consent to participate in this study.

## Author Contributions

JD performed the laboratory steps, statistical analysis, and compiled the study findings as a manuscript. CY performed the sequence data processing and edited the manuscript. PYT and ZKM contributed to collect the samples. XYS contributed to the study concept and design, supervision of laboratory steps, and manuscript preparation and revision. SGZ contributed to the study concept and design, overall supervision, clinical management, and revised the manuscript. All authors contributed to the article and approved the submitted version.

## Funding

The authors appreciate the financial support from the National Natural Science Foundation of China (grant number: 81801037), the Beijing Municipal Science and Technology Commission (grant number: Z181100001618015), and the Ministry of Science and Technology of the People’s Republic of China (grant number: 2018FY101005). 

## Conflict of Interest

The authors declare that the research was conducted in the absence of any commercial or financial relationships that could be construed as a potential conflict of interest.

## Publisher’s Note

All claims expressed in this article are solely those of the authors and do not necessarily represent those of their affiliated organizations, or those of the publisher, the editors and the reviewers. Any product that may be evaluated in this article, or claim that may be made by its manufacturer, is not guaranteed or endorsed by the publisher.
