# Supplementary figures and images for: Potential Roles of the Free Salivary Microbiome Dysbiosis in Periodontal Diseases
Source: Front Cell Infect Microbiol. 2021 Sep 22;11:711282. doi: 10.3389/fcimb.2021.711282 (PMC8493099; doi:10.3389/fcimb.2021.711282)

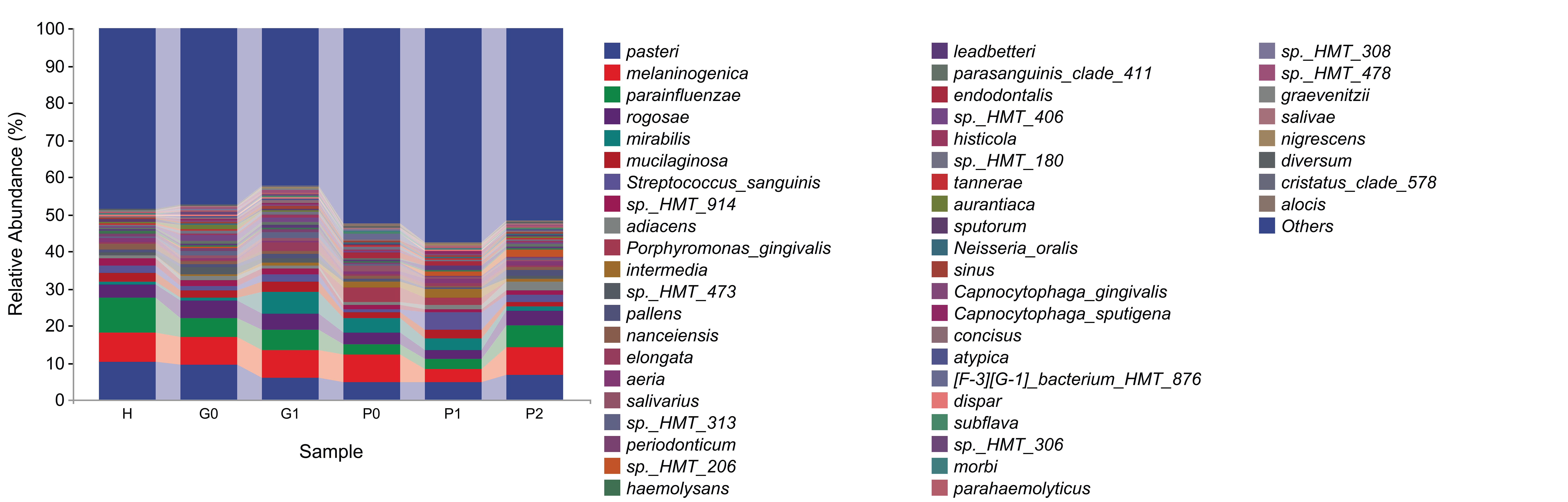

Supplement: Supplementary file 2 [file Image_2.jpeg]

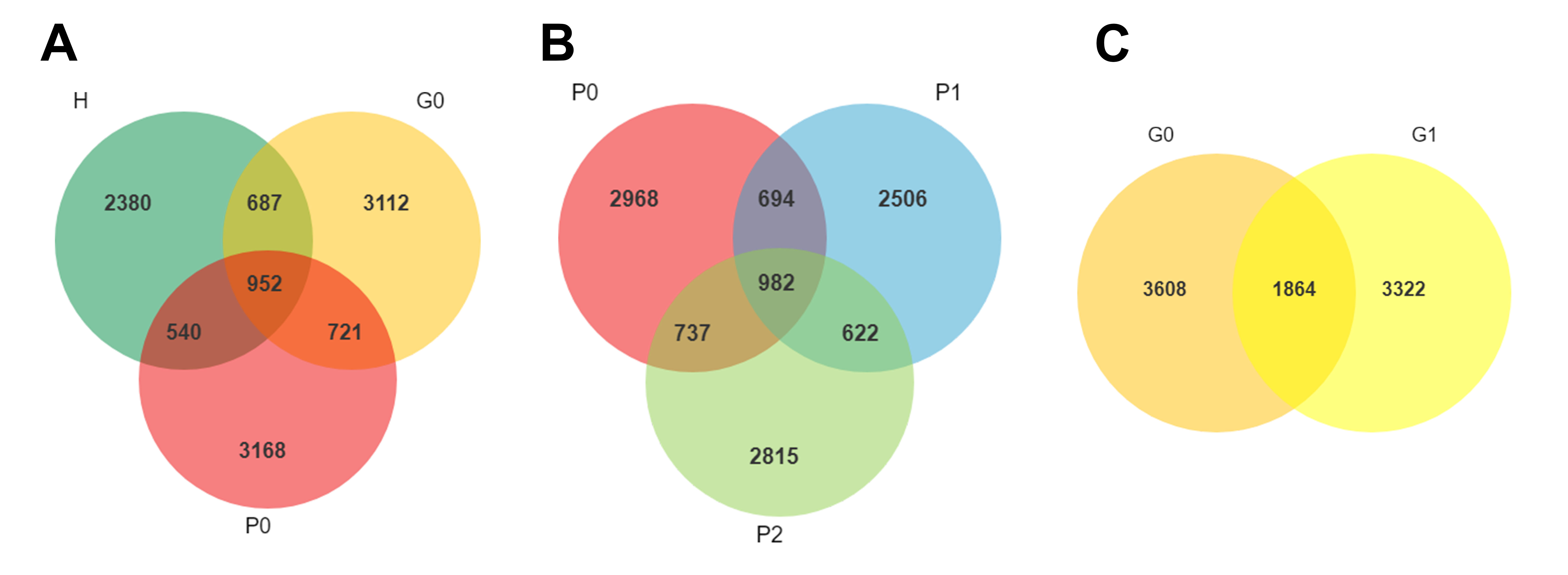

Supplement: Supplementary file 3 [file Image_3.jpeg]
