# Supplementary material for: Potential Roles of the Free Salivary Microbiome Dysbiosis in Periodontal Diseases
Source: Front Cell Infect Microbiol. 2021 Sep 22;11:711282. doi: 10.3389/fcimb.2021.711282 (PMC8493099; doi:10.3389/fcimb.2021.711282)
Supplement: Supplementary file 5 [file Table_1.doc]

**Table 1: Clinical periodontal indices of group P**

| Clinical parameters | T0 | T1 | T2 |
| --- | --- | --- | --- |
| PD(mm) | 2.78±0.69 | 2.71±0.48 | 2.20±0.27*† |
| BI | 2.42±0.84 | 2.27±0.68 | 1.66±0.51*† |
| BOP(%) | 57.40±24.01 | 51.39±21.54 | 31.09±13.65*† |

Full-mouth periodontal parameters of the individuals with periodontitis (group P) over the experimental period (n=15, male =6, female=9). Mean age: 39.80±11.08 years.

*P*-value: paired-samples t-test.

* Significantly different from T0.

† Significantly different from T1.

**Table 2: Clinical periodontal indices of group G**

| Clinical parameters | T0 | T1 |
| --- | --- | --- |
| PD(mm) | 1.94±0.27 | — |
| BI | 1.27±0.46 | 1.05±0.46 |
| BOP(%) | 28.89±1.86 | 17.36±11.13# |

Full-mouth periodontal parameters of the individuals with periodontitis (group P) over the experimental period (n=15, male =5, female=10). Mean age: 33.67±11.20 years.

*P*-value: paired-samples t-test.

# Significantly different from T0.

**Table 3: Top-30 OTUs in grouping the six groups by random forest analysis**

| OTU | Taxon |
| --- | --- |
| OTU_7 | *Porphyromonas gingivalis* |
| OTU_154 | *Streptococcus gordonii* |
| OTU_71 | *Prevotella nigrescens* |
| OTU_13 | *Porphyromonas endodontalis* |
| OTU_22 | *Streptococcus sanguinis* |
| OTU_41 | *Filifactor alocis* |
| OTU_114 | *Tannerella forsythia* |
| OTU_9138 | *Streptococcus unclassified_Streptococcus* |
| OTU_37 | *Prevotella nanceiensis* |
| OTU_3 | *Lautropia mirabilis* |
| OTU_16 | *Prevotella melaninogenica* |
| OTU_277 | *Prevotella nanceiensis* |
| OTU_258 | *Prevotella melaninogenica* |
| OTU_12 | *Capnocytophaga gingivalis* |
| OTU_34 | *Granulicatella adiacens* |
| OTU_332 | *Neisseria unclassified_Neisseria* |
| OTU_98 | *Treponema denticola* |
| OTU_30 | *Capnocytophaga sputigena* |
| OTU_162 | *Oribacterium sinus* |
| OTU_366 | *Granulicatella adiacens* |
| OTU_14 | *Prevotella intermedia* |
| OTU_562 | *Neisseria unclassified_Neisseria* |
| OTU_207 | *Streptococcus cristatus_clade_578* |
| OTU_47 | *Peptostreptococcaceae_[XI][G-6] [XI][G-6]_nodatum* |
| OTU_469 | *Streptococcus unclassified_Streptococcus* |
| OTU_199 | *Streptococcus parasanguinis_clade_411* |
| OTU_229 | *Streptococcus unclassified_Streptococcus* |
| OTU_980 | *Neisseria unclassified_Neisseria* |
| OTU_8557 | *Prevotellaceae Prevotella veroralis* |
| OTU_27 | *Campylobacter concisus* |

**Figure legends**

**Appendix Fig.1:** Rarefaction analysis of the species richness of the salivary microbiota in each sample.

**Appendix Fig.2:** The overall structure of the salivary microbiota by species in different groups of saliva samples. The legend exhibited the top-50 taxa in terms of the mean relative abundance.

**Appendix Fig.3:** The number of shared and unique OTUs in each group.

**Appendix Fig.4:** Predictive function analysis by PICRUSt2 based on KEGG database of each group.
